# Clinical and Procedural Outcomes of IVUS-Guided vs. Angiography-Guided CTO-PCI: A Systematic Review and Meta-Analysis

**DOI:** 10.3390/jcm12154947

**Published:** 2023-07-27

**Authors:** Giuseppe Panuccio, Youssef S. Abdelwahed, Nicole Carabetta, Nadia Salerno, David Manuel Leistner, Ulf Landmesser, Salvatore De Rosa, Daniele Torella, Gerald S. Werner

**Affiliations:** 1 Department of Medical and Surgical Sciences, Magna Graecia University, 88100 Catanzaro, Italy; nicole.carabetta95@gmail.com; 2 Department of Cardiology, Angiology and Intensive Care Medicine, Deutsches Herzzentrum der Charité Berlin, 12200 Berlin, Germany; youssef.abdelwahed@charite.de (Y.S.A.); ulf.landmesser@charite.de (U.L.); 3 DZHK (German Centre for Cardiovascular Research), 10785 Berlin, Germany; 4 Department of Experimental and Clinical Medicine, Magna Graecia University, 88100 Catanzaro, Italy; nadia.salerno@unicz.it (N.S.); dtorella@unicz.it (D.T.); 5 Department of Medicine, Cardiology, Goethe University Hospital, 60596 Frankfurt, Germany; david.leistner@kgu.de; 6 German Center for Cardiovascular Research, Partner Site RheinMain, 60590 Frankfurt, Germany; 7 Berlin Institute of Health (BIH), 10178 Berlin, Germany; 8 Medizinische Klinik I Klinikum Darmstadt, 64283 Darmstadt, Germany; gerald.s.werner@gmail.com

**Keywords:** chronic total occlusions, intravascular ultrasound, complex PCI, intravascular imaging, IVUS

## Abstract

Chronic total occlusions (CTO) in coronary angiographies present a significant challenge nowadays. Intravascular ultrasound (IVUS) is a valuable tool during CTO-PCI, aiding in planning and achieving procedural success. However, the impact of IVUS on clinical and procedural outcomes in CTO-PCI remains uncertain. This meta-analysis aimed to compare IVUS-guided and angiography-guided approaches in CTO-PCI. The study included five studies and 2320 patients with stable coronary artery disease (CAD) and CTO. The primary outcome of major adverse cardiac events (MACE) did not significantly differ between the groups (*p* = 0.40). Stent thrombosis was the only secondary clinical outcome that showed a significant difference, favoring the IVUS-guided approach (*p* = 0.01). Procedural outcomes revealed that IVUS-guided procedures had longer stents, larger diameters, and longer procedure and fluoroscopy times (*p* = 0.007, *p* < 0.001, *p* = 0.03, *p* = 0.002, respectively). Stent number and contrast volume did not significantly differ between the approaches (*p* = 0.88 and *p* = 0.33, respectively). In summary, routine IVUS use did not significantly improve clinical outcomes, except for reducing stent thrombosis. Decisions in CTO-PCI should be individualized based on patient characteristics and supported by a multi-parametric approach.

## 1. Introduction

Coronary artery disease (CAD) is the third major global cause of mortality [1,2]. Despite the advancement of percutaneous coronary intervention (PCI), the presence of chronic total occlusions (CTO) at coronary angiography—ranging 16–18%—still represents a challenge nowadays, both from a technical and clinical point of view [3,4]. Over the years, the development of new materials, antegrade and retrograde approach techniques, and the increased expertise of the operators brought close to 90% the current success rate of CTO-PCI [5,6]. Among the available tools and devices, intravascular ultrasound (IVUS) can be very helpful during PCI, and more so when dealing with a CTO. The benefit of IVUS in CTO has been demonstrated for many years [7,8]. Its applications vary from assessing plaque composition, understanding mechanisms underlying stent failure, selecting the most adequate debulking method to prepare lesions (angioplasty, atherectomy or lithotripsy), choosing the best stent size, and confirming optimal stent expansion, as well as excluding malapposition or stent fracture [9,10]. The primary role of IVUS in PCI was recently underlined by the RENOVATE-COMPLEX-PCI trial, which showed that in patients with complex CAD, intravascular imaging-guided PCI significantly reduced the composite endpoint of death from cardiac causes, target vessel-related myocardial infarction, or clinically driven target vessel revascularization [11]. However, despite the fact that the role of IVUS in reducing clinical outcomes in complex CAD seems to be clear [12], the effects of IVUS on clinical and procedural outcomes in CTO-PCI still remain uncertain. IVUS is a crucial imaging device in CTO-PCI, and its applications are many, both in the antegrade and retrograde approaches. In more details, when a side branch is large enough to fit a probe, IVUS can show plaque morphology and thus solve the problem of the “ambiguous proximal cap”, one of the most challenging findings in CTO-PCI [13]. Also, IVUS can simplify re-entry techniques, provide real-time confirmation of the location of a second wire in the distal true lumen, and identify the best re-entry point, avoiding calcific segments and segments with excessive gap between the false and true lumen [14]. In the retrograde approach, IVUS is helpful to identify the position of both antegrade and retrograde wires (intraplaque vs. subintimal) and to guide their connection by balloon inflation. In ostial CTOs, a retrograde approach is frequently necessary. In this scenario, IVUS could help to avoid inadvertent subintimal wiring in the left main coronary artery or along the aortic wall, creating dangerous dissections. Regarding stent implantation, IVUS is crucial to determine the real vessel size (media-to-media) and identify any areas of negative remodeling where stent expansion might result in vessel rupture [15]. Notwithstanding all these uses of IVUS in CTO-PCI, its impact on clinical and procedural outcomes is uncertain. Consequently, we performed the present meta-analysis to compare the clinical and procedural outcomes of an IVUS-guided versus angiography-guided approach to CTO-PCI.

## 2. Materials and Methods

The Cochrane Collaboration and PRISMA criteria were followed for conducting this meta-analysis [16,17].

### 2.1. Research

Scientific literature was systematically searched for studies reporting clinical and procedural outcomes for different strategies of revascularization (IVUS-guided versus angiography-guided) in patients undergoing CTO-PCI. Articles were searched for on the following public databases: PubMed (https://pubmed.ncbi.nlm.nih.gov/) and Cochrane Library (https://www.cochranelibrary.com/) accessed until 22 March 2023). We used the following keywords: CTO or chronic total occlusions, (pci or PTCA), IVUS.

### 2.2. Study Selection with Inclusion/Exclusion Criteria

To find qualifying studies, two co-authors (GP, SDR) independently reviewed search records. Divergences were settled by discussion and agreement. Studies were considered eligible if they met all of the following inclusion criteria: (a) any clinical study in which an IVUS-guided CTO-PCI was compared to an angiography-guided CTO-PCI approach; (b) the clinical scenario was CAD with evidence of a least one CTO; and (c) clinical and procedural outcomes were reported. Exclusion criteria were as follows: clinical scenarios different from stable CAD with evidence of CTOs; studies in which intravascular imaging performed was not IVUS; editorials or review articles; case reports or case series; meta-analyses; clinical and procedural outcomes not reported. Data extraction was carried out by the same co-authors (GP, SDR).

### 2.3. Outcomes

The primary clinical outcome was the incidence of major adverse cardiac events (MACE), defined as a composite of cardiovascular (CV) death, non-fatal myocardial infarction (MI), and target vessel revascularization (TVR) at the longest follow-up available. Secondary clinical outcomes were cardiovascular death, all-cause mortality, MI, TVR, target lesion revascularization (TLR) and stent thrombosis. Total stent length, stent diameter, total number of stents, procedure time, fluoroscopy time, and contrast volume were also analyzed as procedural outcomes.

### 2.4. Evaluation of Study Quality

Two co-authors evaluated study quality (GP, SDR). Divergences were handled by discussing and coming to a consensus. According to the ROBINS-II tool, the risk of numerous forms of bias was assessed: randomization process, deviation from the intended intervention, missing outcome data, outcomes’ measurement, and selection of the reported results [18].

### 2.5. Statistical Analysis

Discrete data were reported as percentages, whereas continuous values were summarized as mean and standard deviation. The Mantel–Haenszel random effects model was used to compute summary effect sizes, and the findings of clinical outcomes were shown as risk ratios (RR) with 95% confidence intervals (95% CI), while differences regarding continuous procedural outcomes were expressed as standardized mean difference (SMD) with 95% CI. To determine the appropriate sample size to demonstrate or reject a 20% relative risk reduction with IVUS-guided CTO PCI with an alpha error of 5% and a statistical power of 80%, trial sequential analysis (TSA) was conducted. Meta-analysis was performed using OpenMetaAnalyst 10 (Brown University, Providence, Rhode Island) and Comprehensive Meta-analysis Software (Biostat Inc.14 North Dean Street Englewood, NJ, USA). The TSA computer application (Version 0.9.5.10 Beta. The Copenhagen Trial Unit, Centre for Clinical Intervention Research, The Capital Region, Copenhagen University Hospital—Rigshospitalet, 2021) was used to carry out TSA. Study bias was evaluated by examining funnel plots graphically and using Egger’s and Begg’s tests. Heterogeneity of studies was assessed with Cochran’s Q test, and it was measured using the Inconsistency index (I^2^). PROSPERO, international prospective register of systematic reviews, has the analytic procedure listed (PROSPERO record ID: CRD42023410342).

## 3. Results

### 3.1. Selected Studies and Baseline Characteristics

**Figure 1:**
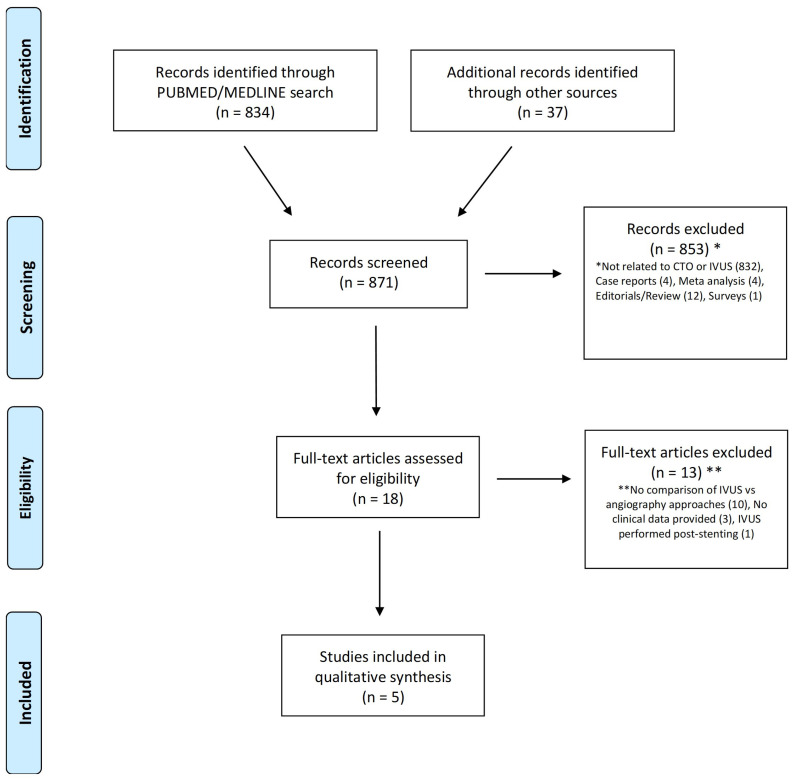
Prisma flow-chart of included trials.

Five studies (2320 patients with stable complex CAD and evidence of CTO) were included in this analysis out of the 871 studies that were screened (Figure 1) [19,20,21,22,23]. Two studies were randomized controlled trials (RCTs), while three were observational studies. The average age was 64.1 ± 2.9 years. All patients were hospitalized for CTO-PCI, and the majority of them had significant cardiovascular risk profiles. In all the included studies, IVUS was performed for stent implantation and optimization. Furthermore, in the AIR-CTO trial, IVUS was also used to confirm the wire’s position in the true lumen. In fact, IVUS provides useful information to guide the proximal puncture of the proximal cap, particularly when substantial calcifications are present. Table 1 lists study baseline characteristics, while Table 2 lists the specifics for the single studies.

### 3.2. Primary Clinical Outcome

**Figure 2:**
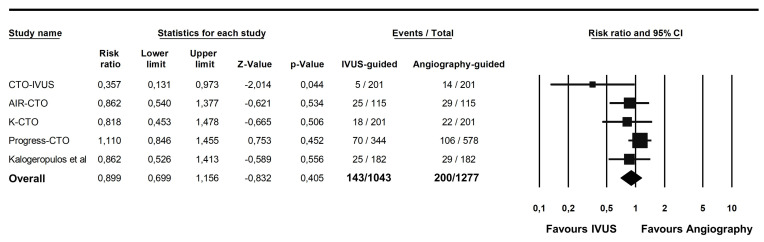
Forest plot of the primary endpoint [23].

From the 2320 patients included in the primary analysis, 143 patients (13.7%) presented with the primary outcome of MACE in the IVUS-guided group, versus 200 patients (15.6%) in the angiography-guided group (RR 0.89; 95% CI 0.69–1.15; *p* = 0.40; Figure 2). TSA demonstrated that the size of the effect was large enough to provide a reliable and solid result (Z curve coefficient 0.83; Supplementary Figure S1). Subgroup analysis for study design showed that the results were consistent across RCTs and observational studies (Supplementary Figure S2).

### 3.3. Secondary Clinical Outcomes

**Figure 3:**
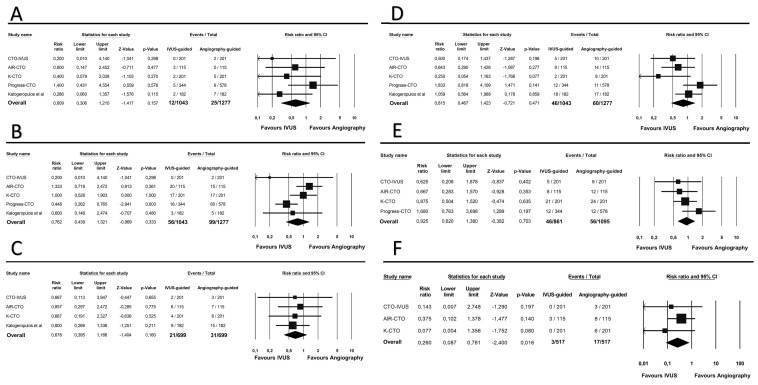
Forest plot of the secondary clinical endpoints [23]. (**A**) CV death; (**B**) myocardial infarction; (**C**) all-cause mortality; (**D**) target vessel revascularization; (**E**) target lesion revascularization; (**F**) stent thrombosis.

A CV death was registered in 12 patients (1.1%) in the IVUS-guided group and in 25 patients (1.9%) in the angiography-guided group (RR 0.60; 95% CI 0.30–1.21; *p* = 0.15). MI occurred in 56 patients (5.3%) in the IVUS-guided group, as compared to 99 patients (7.7%) in the angiography-guided group (RR 0.76; 95% CI 0.43–1.32; *p* = 0.33). All-cause mortality occurred in 21 patients (3.0%) in the IVUS-guided group, versus 31 patients (4.4%) in the angiography-guided arm (RR 0.67; 95% CI 0.39–1.16; *p* = 0.16). TVR was found in 46 patients (4.4%) of the IVUS-guided group, and in 60 patients (4.6%) of the angiography-guided group (RR 0.81; 95% CI 0.46–1.42; *p* = 0.47). TLR occurred in 46 patients (5.3%) in the IVUS-guided group, versus 56 patients (5.1%) in the angiography-guided group (RR 0.92; 95% CI 0.62–1.38; *p* = 0.70). Finally, stent thrombosis incidence was significantly lower in the IVUS-guided group as compared to the angiography-guided group (0.5% vs. 3.2%; RR 0.26; 95% CI 0.08–0.78; *p* = 0.01). All the results of the secondary endpoints are showed in Figure 3A–F. Subgroup analysis of RCTs did not show any relevant difference between different study designs.

### 3.4. Procedural Outcomes

**Figure 4:**
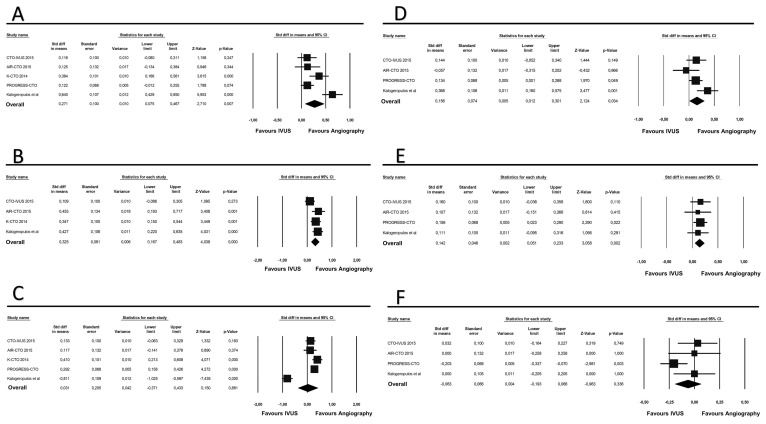
Forest plot of the secondary procedural outcomes [23]. (**A**) stent length; (**B**) stent diameter; (**C**) stent number; (**D**) procedure time; (**E**) fluoroscopy time; (**F**) contrast volume.

Total stent length (SMD 0.27; 95% CI 0.07–0.46; *p* = 0.007) and stent diameter (SMD 0.32; 95% CI 0.08–0.006; *p* < 0.001) were significantly higher in the IVUS-guided group, while no difference was apparent in the mean stent number (SMD 0.03; *p* = 0.88). Since heterogeneity for this outcome was very high (I^2^ = 93%), we performed sensitivity analysis showing a large proportion of heterogeneity attributable to the study by Kalogeropulos et al. In fact, removal of this study yielded a change to moderate heterogeneity (I^2^ = 63%). Conditional analysis without this study showed a higher number of stents implanted in the IVUS-guided group (SMD 0.25; 95% CI 0.12–0.37; *p* < 0.001) (Supplementary Figure S3). In addition, the IVUS-guided approach was associated with longer procedure time (SMD 0.15; 95% CI 0.01–0.30; *p* = 0.03) and fluoroscopy time (SMD 0.14; 95% CI 0.05–0.23; *p* = 0.002). Lastly, no difference in mean contrast volume was found between the two approaches (SMD −0.06; 95% CI −0.19–0.06; *p* = 0.33). All the results of the procedural outcomes analysis are depicted in Table 3 and Figure 4A–F.

### 3.5. Study Quality

A low to moderate risk of bias was found with the included studies (Supplementary Figure S4). Heterogeneity was low to moderate, except in the comparison of total number of stents. Excluding a single study (Kalogeropulos et al.) [20] in the quantitative comparison of total number of stents, heterogeneity became moderate for all the outcomes. Graphical assessment of funnel plots did not show severe asymmetries. Both Egger’s and Begg’s tests were concordant with the funnel plots (Supplementary Figures S5–S7).

## 4. Discussion

The use of IVUS during CTO-PCI can solve a number of practical issues in a complex and lengthy procedure. Yet, the general clinical impact of IVUS use in this scenario is difficult to measure. In fact, in a consensus paper published by the European Association of Percutaneous Cardiovascular Interventions, it was emphasized that IVUS guidance compared to angiography could significantly improve clinical outcomes [24]. However, the trials mentioned in this consensus paper included both patients with CTO and non-CTO CAD (bifurcations, long lesions etc.). Therefore, only few studies compared IVUS-guided to angiography-guided CTO-PCI, all with a limited number of patients, thus not allowing for a conclusive definition of IVUS clinical impact. To summon all available evidence, we performed this meta-analysis, showing that a routine use of IVUS in CTO-PCI does not improve clinical outcomes such as MACE, all-cause mortality, MI, or CV mortality, but can significantly reduce rate of stent thrombosis. The latter is probably due to the fact that IVUS allows us to better measure vessel dimensions, cover completely the lesions and reduce the risk of stent malapposition or undersizing, known risk factors of stent thrombosis [25]. In line with our hypothesis, a systematic underestimation of vessel size, often by as much as 1–2 mm, was shown by Park et al. in 58 CTO patients serially re-assessed with IVUS 3 months after the index CTO recanalization. Not surprisingly, stent optimization appeared to be the strongest predictor of long-term success [26]. Our results about procedural outcomes, showing that the use of IVUS is characterized by a significantly higher stent length and stent diameter (probably due to a better measurement of vessel size), also point in the same direction. Our results show that the use of IVUS is associated with an increase in total stent length and mean stent diameter. While the latter result is not surprising at all, the use of longer stents is apparently more difficult to justify and counterintuitive. On the other hand, it should be pointed out that no significant increase in the number of stents used was registered. One of the most useful applications of IVUS when dealing with CTO is the assessment of the proximal cap. In fact, it is very useful both to resolve proximal cap ambiguity or to assess the presence and relevance of calcifications to select the most appropriate interventional strategy, as suggested in the recent EAPCI/EURO4C-PCR consensus [27]. Unfortunately, data on the use of IVUS to resolve the proximal cap and/or assess the calcium burden were not comprehensively reported across the studies. Currently, the largest trial regarding this topic is the PROGRESS-CTO trial [25], which included 922 patients undergoing IVUS- versus angiography-guided CTO-PCI, the results of which are in line with our overall results, both on clinical and procedural aspects. The finding of a significant reduction in the rate of stent thrombosis associated with the use of IVUS is reassuring. In fact, it suggests that the benefits associated with the use of intracoronary imaging are not limited to procedural outcomes but potentially reflect into actual clinical impact. The nature of the present work does not allow us to define the mechanisms underlying this finding. However, it is tempting to speculate that the larger mean stent diameter might have played a role. In addition, the additional information provided by IVUS might have been reflected into a more personalized technique, although this is technically more difficult to capture in traditional clinical studies. Our findings are consistent with recently published studies, showing that IVUS allows for better visualization of vessel size and morphology before stent placement [28]. IVUS guidance is characterized by a significantly higher procedure and fluoroscopy time. This result does not come as a surprise. Possible reasons for the longer times could be both the time needed to perform IVUS runs, and the time used for additional optimizations that would not have been performed without IVUS results. Previous meta-analyses have assessed the overall impact of IVUS in CTO-PCI. Nevertheless, there are significant differences with our work that should be underlined. First, the work from Zhong et al. only included clinical outcomes, without reporting on procedural outcomes [29]. In contrast, procedural data are indeed very relevant for such a complex PCI as for recanalization of CTOs. Second, we included a larger number of studies compared to the metanalysis by Chug et al., which substantially changed the results’ outlook for procedural outcomes [30]. Furthermore, we also included a larger number of analyses (e.g., mean stent diameter). Finally, our meta-analysis is the first to feature a TSA, which can be very useful for a topic where large studies are very difficult to undertake.

### Limitations

The ongoing and rapid advancement of therapeutic methods, interventional techniques, and materials frequently causes research results to become out of date once they are published, as is typical with interventional studies of percutaneous interventions, especially in CTO. Some of the studies included are from 2014 and 2015. Moreover, in the AIR-CTO study, only less then 50% of the patients were treated with a 2nd generation drug eluting stent (DES). Therefore, not all materials and interventional techniques used would reflect the actual state of the art, which may restrict the application of our findings to a typical nowadays scenario. Also, observational studies were included in this meta-analysis, leading to a possible risk of bias. Nevertheless, sensitivity analysis showed that the general outcomes’ outlook was not affected by the exclusion of non RCTs. Not all the studies included reported all data regarding both clinical and procedural outcomes, as well as some baseline characteristics. More in detail, the PROGRESS-CTO trial did not report data regarding all-cause mortality, stent thrombosis, and stent diameter, while the study from Kalogeropulos et al. did not report data regarding stent thrombosis and TLR. Finally, in the K-CTO trial, no data of procedure time, fluoroscopy time, and contrast amount were provided. The overall number of patients included is another drawback of this meta-analysis. In fact, there are limited studies comparing IVUS guidance with angiographic guidance in patients with CTO. Therefore, other randomized controlled trials ongoing (NCT04917432) will provide information about this important topic. The recent RENOVATE-COMPLEX-PCI trial shed new lights on the significant role of intravascular imaging in complex PCI to reduce MACE. In this trial, there were 319 patients whom complex CAD was characterized by CTOs. In the CTO subgroup analysis performed in this trial, the use of intravascular imaging significantly reduced the primary outcome in contrast to angiography guide (HR 0.30; 95% CI 0.13–0.71). As this trial included both IVUS and optical coherence tomography as intravascular imaging device, we could not include those patients in our analysis. However, the results are in line with this meta-analysis and support our findings.

## 5. Conclusions

A routinary use of IVUS to guide CTO-PCI was associated with a significant reduction in stent thrombosis rate. However, no impact on other clinical outcome measure was observed. IVUS guidance was associated with a significant increase in stent length and diameter, as well as procedure and fluoroscopy time. Results of ongoing randomized trials will hopefully provide significant and much awaited insight to this topic. Meanwhile, available data and expert consensus documents suggest that IVUS imaging is crucial in CTO-PCI to maximize obtain procedural success, as it provides relevant information to inform the selection of the most appropriate technique upfront and to guide the procedure in itinere. Therefore, despite that IVUS imaging is crucial in CTO-PCI to obtain procedural success, decisions should be tailored to the patient’s profile and supported by a multi-parametric approach.

## Figures and Tables

**Table 1:** Study baseline characteristics.

	K-CTO	CTO-IVUS	Air-CTO	Progress-CTO	Kalogeropoulos et al.
Year	2014	2015	2015	2020	2021
Study type	Observational	Randomized Controlled Trial	Randomized controlled trial	Observational	Observational
Sample Size	402 IG: 201 AG: 201	402 IG: 201 AG: 201	230 IG: 115 AG: 115	922 IG: 344 AG: 578	364 IG: 182 AG: 182
Follow-Up (Years)	2	1	1	1	4
Primary Endpoint	Definite or probable stent thrombosis	Cardiac Death	In-stent late lumen loss (LLL)	CD, MI, TVR	All cause death, CD, MI, TVR
Procedural Success	NR	IG 99 AG 98	IG 91 AG 68	NR	NR
Retrograde Approach (%)	NR	IG: 7 AG 9.5	IG: 10.4 AG: 19.1	IG: 28.8 AG: 21.4	25.5 IG: 30.2 AG: 20.9
Anterograde Approach (%)	NR	IG: 93 AG: 90.5	IG: 89.6 AG: 80.9	IG: AWE 53.5 ADR:17.4 AG: AWE 57.1 ADR: 19.8	IG: AWE 60.4 ADR: 9.3 AG: AWE 69.2 ADR: 9.9
Second-Generation DES (%)	100	100	IG 27.8 AG 20.0	NR	100

CD: cardiac death; MI: myocardial infarction; TVR: target vessel revascularization. AWE: antegrade wire escalation; ADR: antegrade dissection re-entry. IG: intravascular ultrasound-guided group; AG: angiography-guided group.

**Table 2:** Baseline patients’ characteristics.

Study	Year	Age	Diabetes (%)	HTN (%)	3 Vessel Disease (%)	DES (%)	Male Sex (%)	CKD (%)	Femoral Access (%)	Radial Access (%)	LVEF (%)	LAD CTO(%)
K-CTO	2014	62	30.5	59	31	100	77	NR	NR	NR	NR	39.0
CTO-IVUS	2015	61.2	34.3	63.2	34.8	100	80.6	NR	73.1	NR	56.8	44.3
AIR-CTO	2015	66.5	28.3	72.6	NR	100	84.35	NR	11.7	42.1	NR	40.4
PROGRESS-CTO	2020	64.8	51	90.9	NR	99.4	82.5	NR	NR	58.7	50.8	28.1
KALOGEROPOULOS ET AL.	2021	66.2	22.2	70.0	NR	100	82.1	18.4	NR	NR	Good (>55%) IG 71.4 AG 69.8 Mildy reduced (30-49%) IG 24.2 AG 27.5 Reduced (<30%) IG 2.7 AG 2.7	26.6

HTN: Hypertension; DES: Drug Eluting Stent; IG: IVUS-guided; AG: Angiography-guided; NR: Not reported.

**Table 3:** Procedural outcomes data. IG: IVUS-guided; AG: angiography-guided.

	K-CTO		CTO-IVUS		Air-CTO		Progress-CTO		Kalogeropoulos et al.	
	IG	AG	IG	AG	IG	AG	IG	AG	IG	AG
Stent length	44.9 ± 21.2	37.3 ± 20.6	43.6 ± 18.7	41.5 ± 17.6	55.0 ± 23.0	52.0 ± 25.0	74.5 ± 47.0	68.9 ± 45.3	60.0 ± 39.4	38.0 ± 28.5
Stent diameter	2.9 ± 0.3	2.8 ± 0.3	2.9 ± 0.5	2.8 ± 0.4	3.0 ± 0.4	2.8 ± 0.3	NR	NR	3.5 ± 0.7	3.2 ± 0.3
Stent number	1.7 ± 0.7	1.4 ± 0.6	1.7 ± 0.8	1.6 ± 0.7	1.6 ± 0.9	1.5 ± 0.8	2.3 ± 0.74	2.0 ± 1.4	2.4 ± 0.7	3.0 ± 0.7
Procedure time (min)	NR	NR	95.0 ± 50.0	88.0 ± 47.0	87.0 ± 48.0	90.0 ± 57.0	136.0 ± 80.0	126.0 ± 71.0	138.0 ± 98.2	108.0 ± 60.7
Fluoroscopy time (min)	NR	NR	41.0 ± 26.0	37.0 ± 24.0	77.0 ± 69.0	70.0 ± 61.0	49.4 ± 32.0	44.7 ± 28.9	36.7 ± 26.3	33.9 ± 24.2
Contrast volume (mL)	NR	NR	299.0 ± 128.0	295 ± 123.0	293 ± 141.0	293.0 ± 136.0	216.0 ± 107.0	240 ± 124.0	250.0 ± 88.0	250.0 ± 97.2

## Data Availability

The datasets used and/or analyzed during the current study are available from the corresponding author on reasonable request.

## Supplementary Materials

The following supporting information can be downloaded at: https://www.mdpi.com/article/10.3390/jcm12154947/s1, Figure S1: Trial sequential analysis (TSA) for the primary endpoint; Figure S2: Subgroup analysis of RCTs for the primary endpoint. Figure S3: Conditional analysis of total number of stents without the study from Kalogeropulos et al, showing a higher number of stents implanted in the IVUS-guided group; Figure S4: Traffic light plot for evaluation of risk of bias; Figure S5: Funnel plot of the primary endpoint (MACE); Figure S6: Funnel plots of all-cause mortality (A), CV death (B), MI (C), TVR (D), TLR (E) and stent thrombosis (F). Figure S7: Funnel plots of stent length (A), stent diameter (B), number of stents (C), procedure time (D), fluoroscopy time (E) and contrast volume (F).
