# Supplementary material for: Clinical and Procedural Outcomes of IVUS-Guided vs. Angiography-Guided CTO-PCI: A Systematic Review and Meta-Analysis
Source: J Clin Med. 2023 Jul 27;12(15):4947. doi: 10.3390/jcm12154947 (PMC10419599; doi:10.3390/jcm12154947)
Supplement: Supplementary file 1 [file jcm-12-04947-s001.zip › Supplementary data - R1 .pptx]

## Slide 1
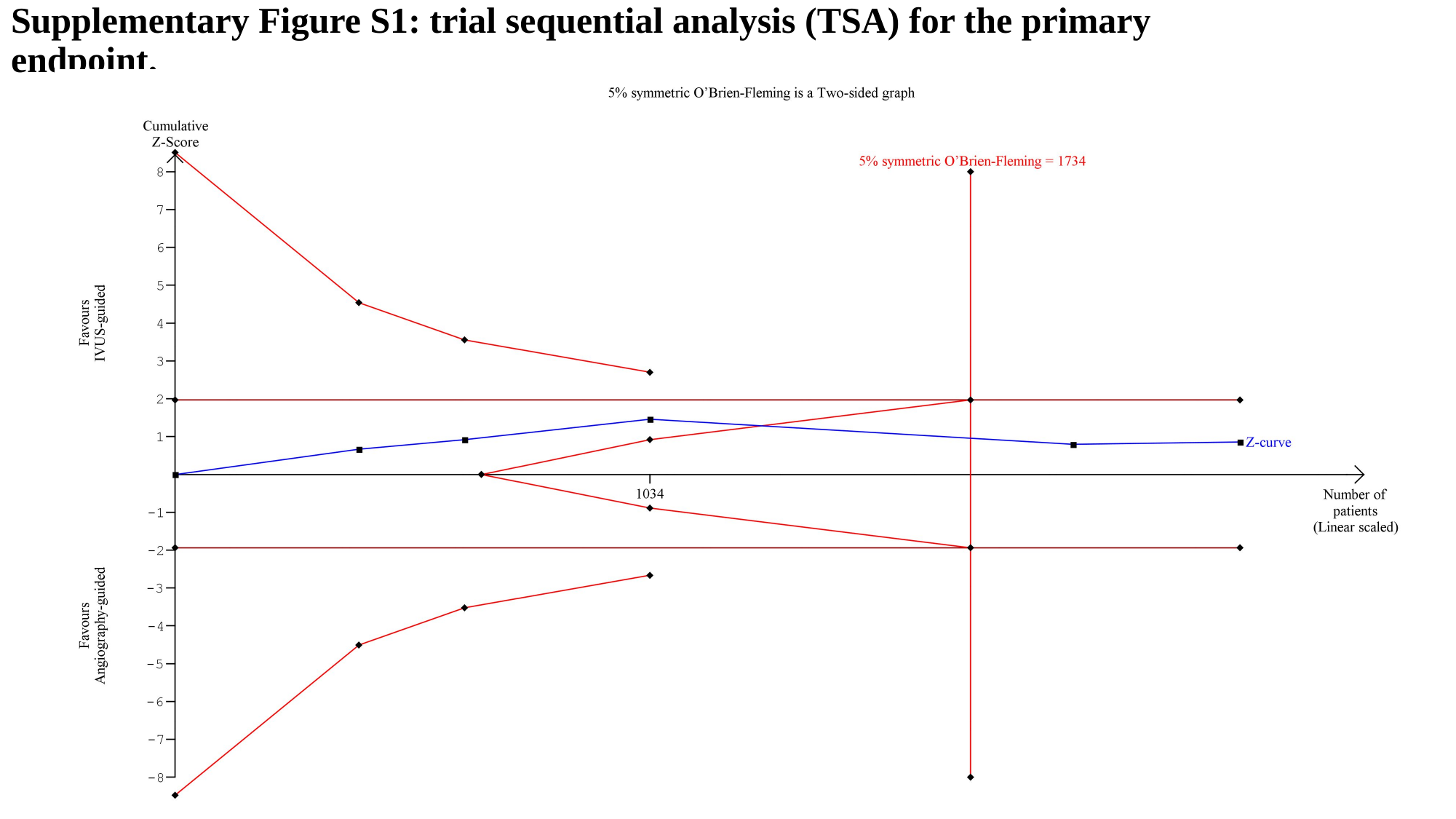

# Supplementary Figure S1: trial sequential analysis (TSA) for the primary endpoint.

## Slide 2
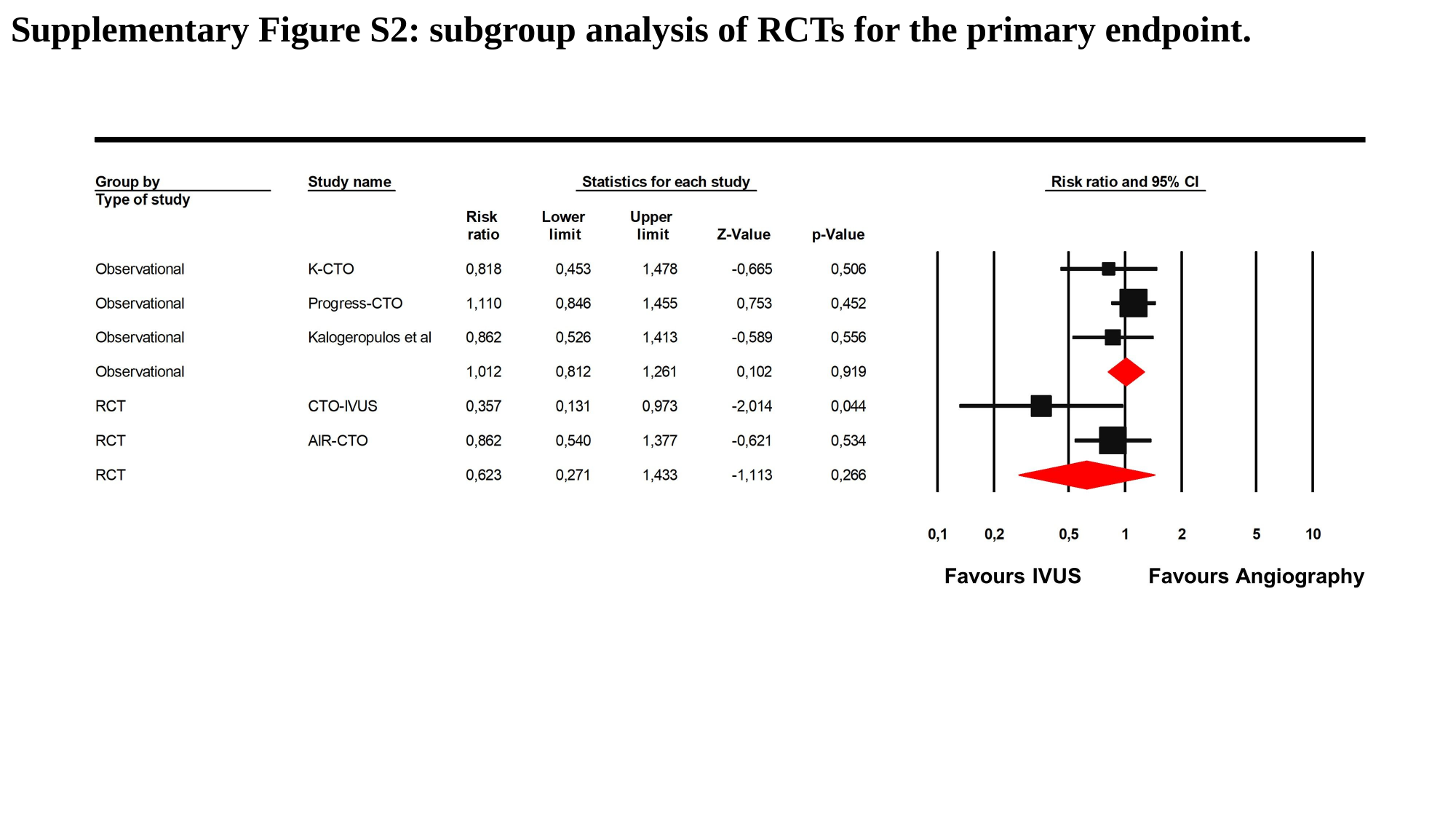

Supplementary Figure S2: subgroup analysis of RCTs for the primary endpoint.

## Slide 3
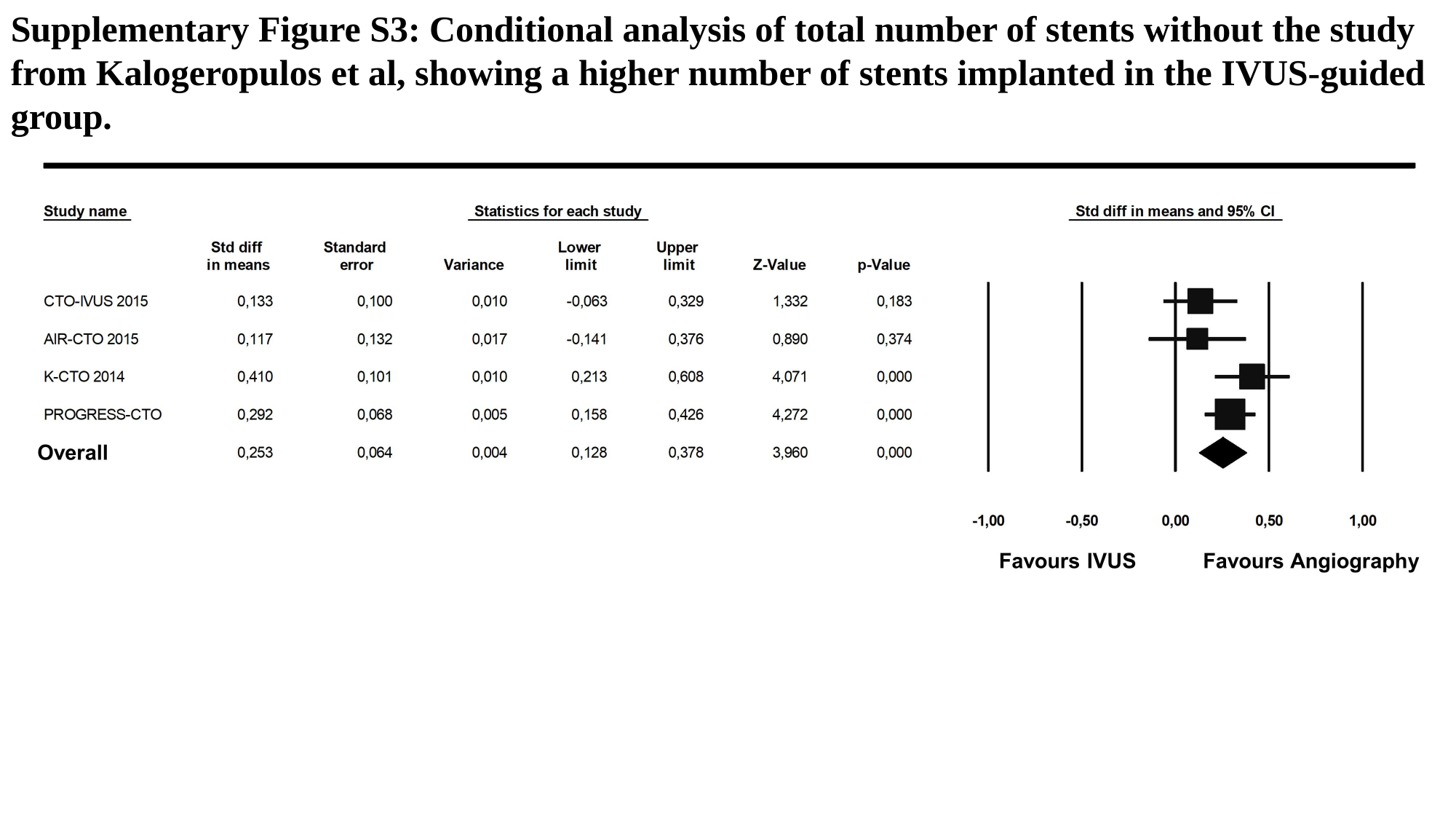

Supplementary Figure S3: Conditional analysis of total number of stents without the study from Kalogeropulos et al, showing a higher number of stents implanted in the IVUS-guided group.

## Slide 4
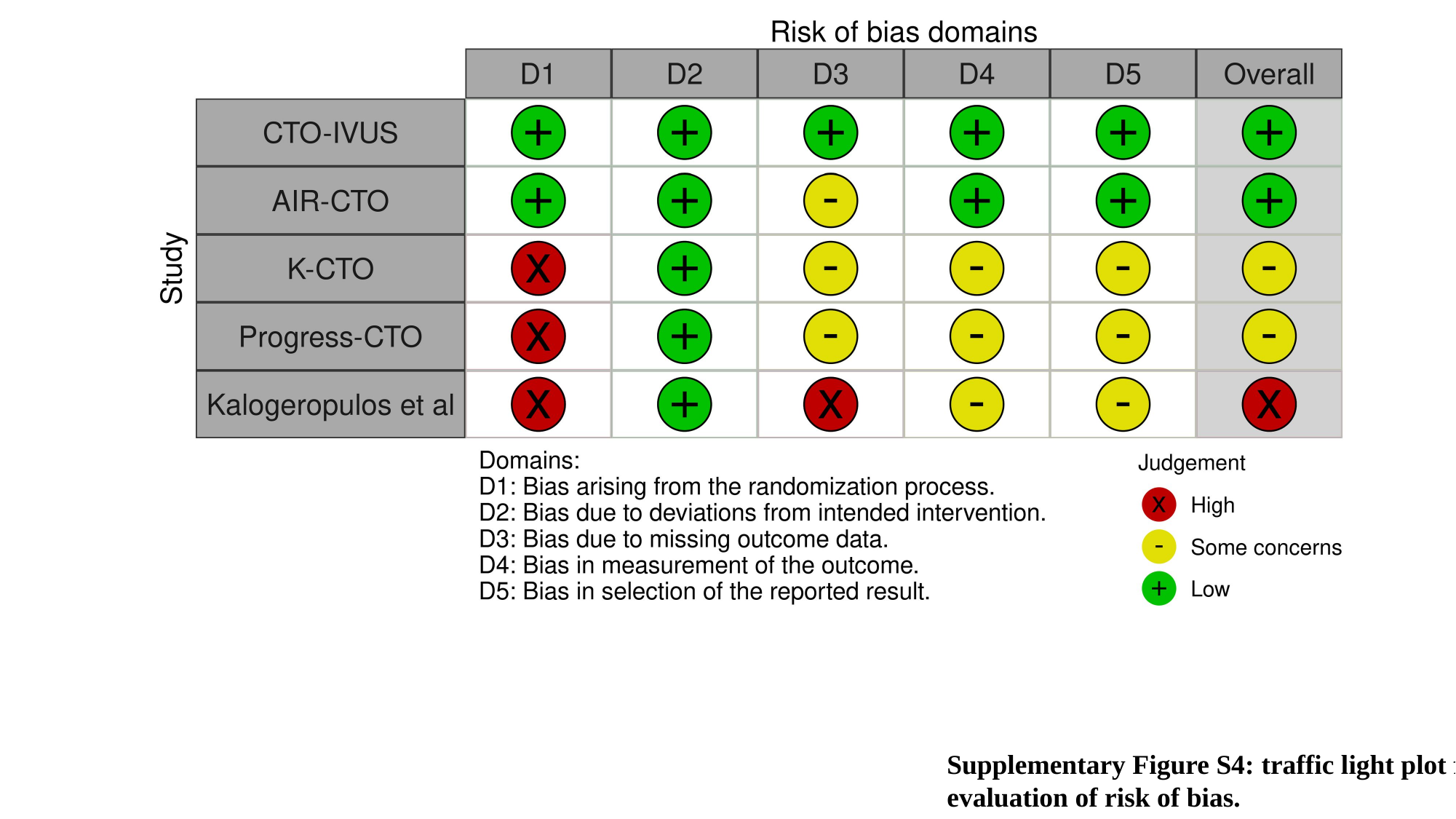

Supplementary Figure S4: traffic light plot for evaluation of risk of bias.

## Slide 5
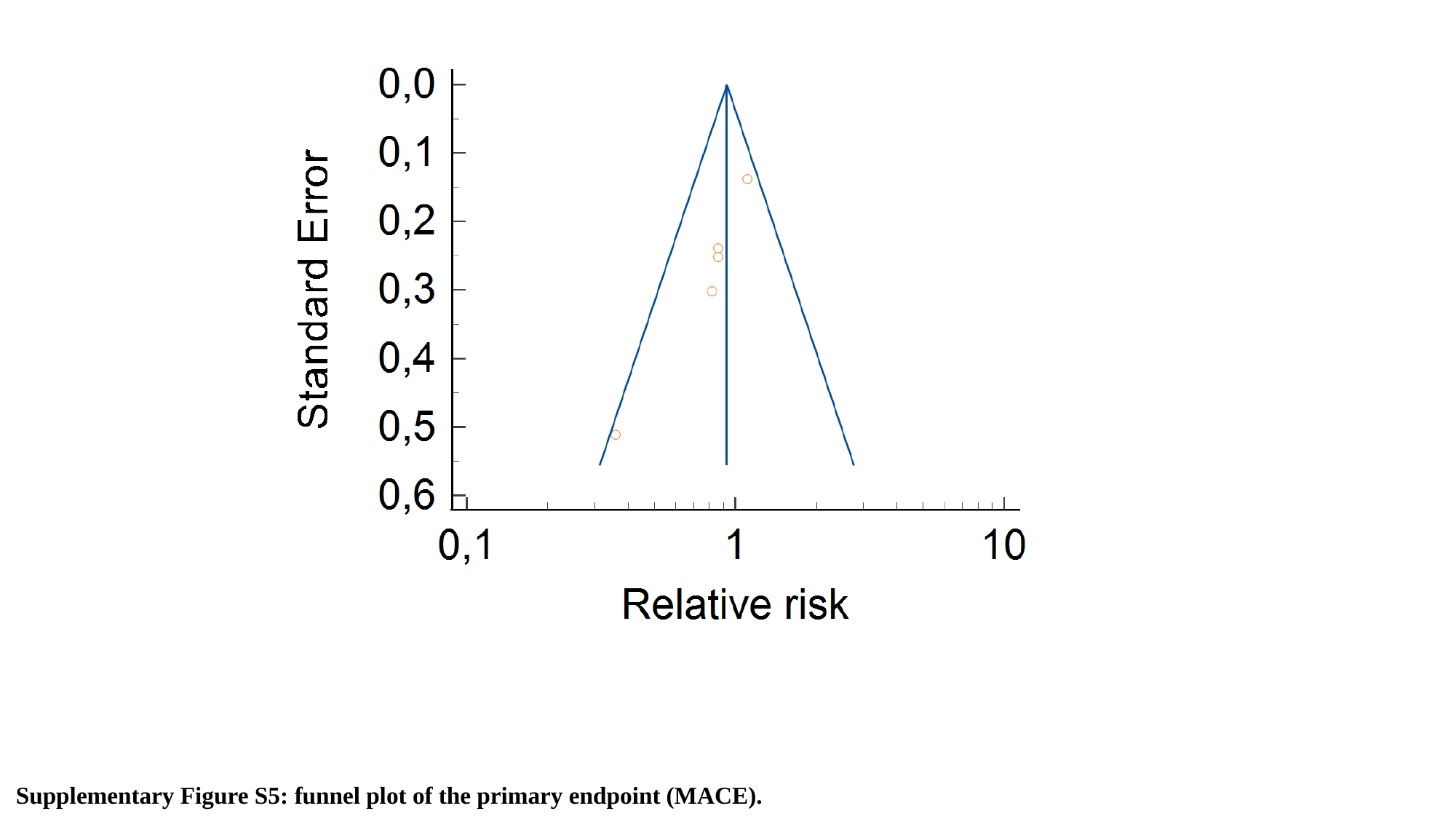

Supplementary Figure S5: funnel plot of the primary endpoint (MACE).

## Slide 6
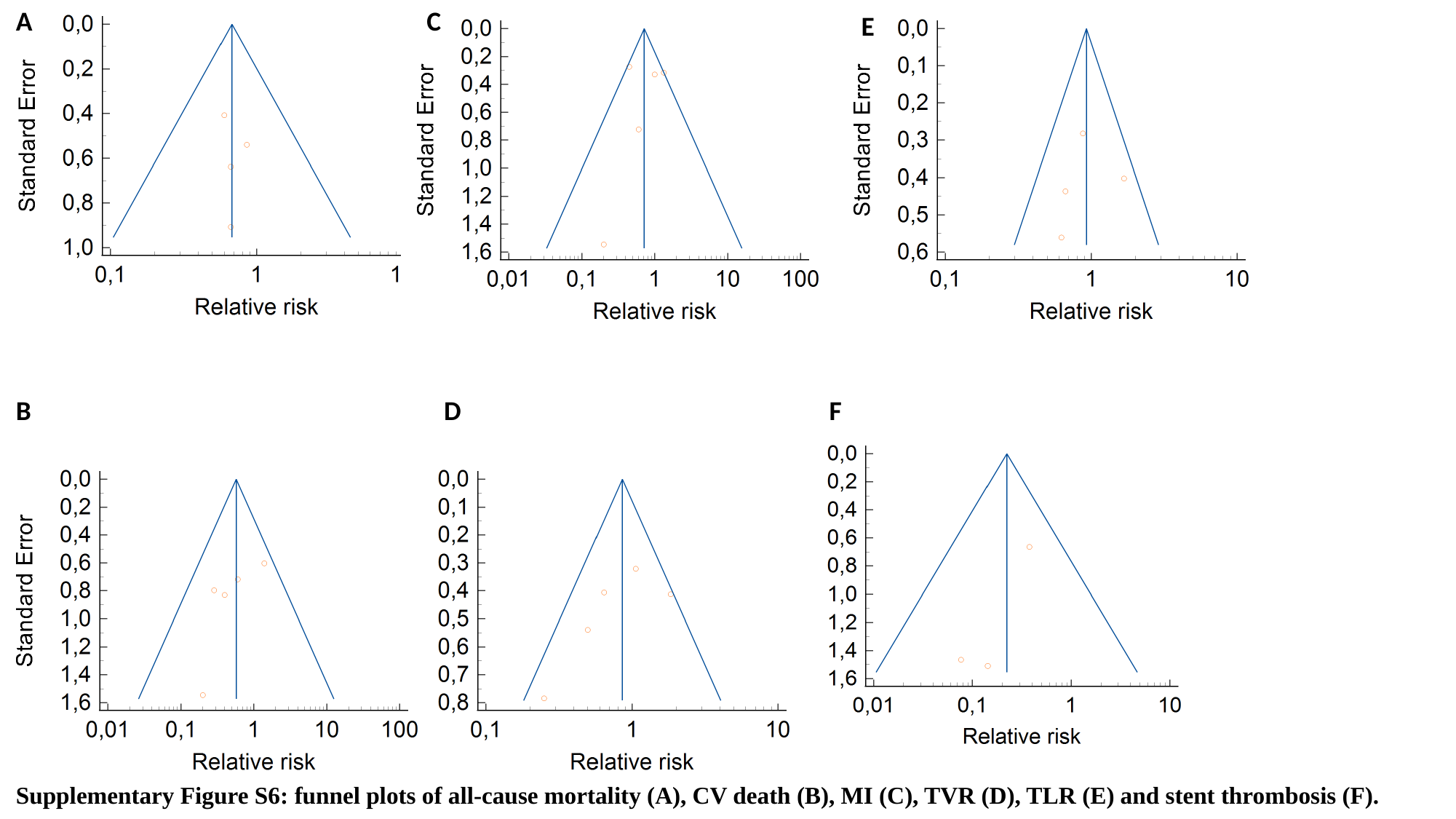

A
C
E
B
D
F
Supplementary Figure S6: funnel plots of all-cause mortality (A), CV death (B), MI (C), TVR (D), TLR (E) and stent thrombosis (F).

## Slide 7
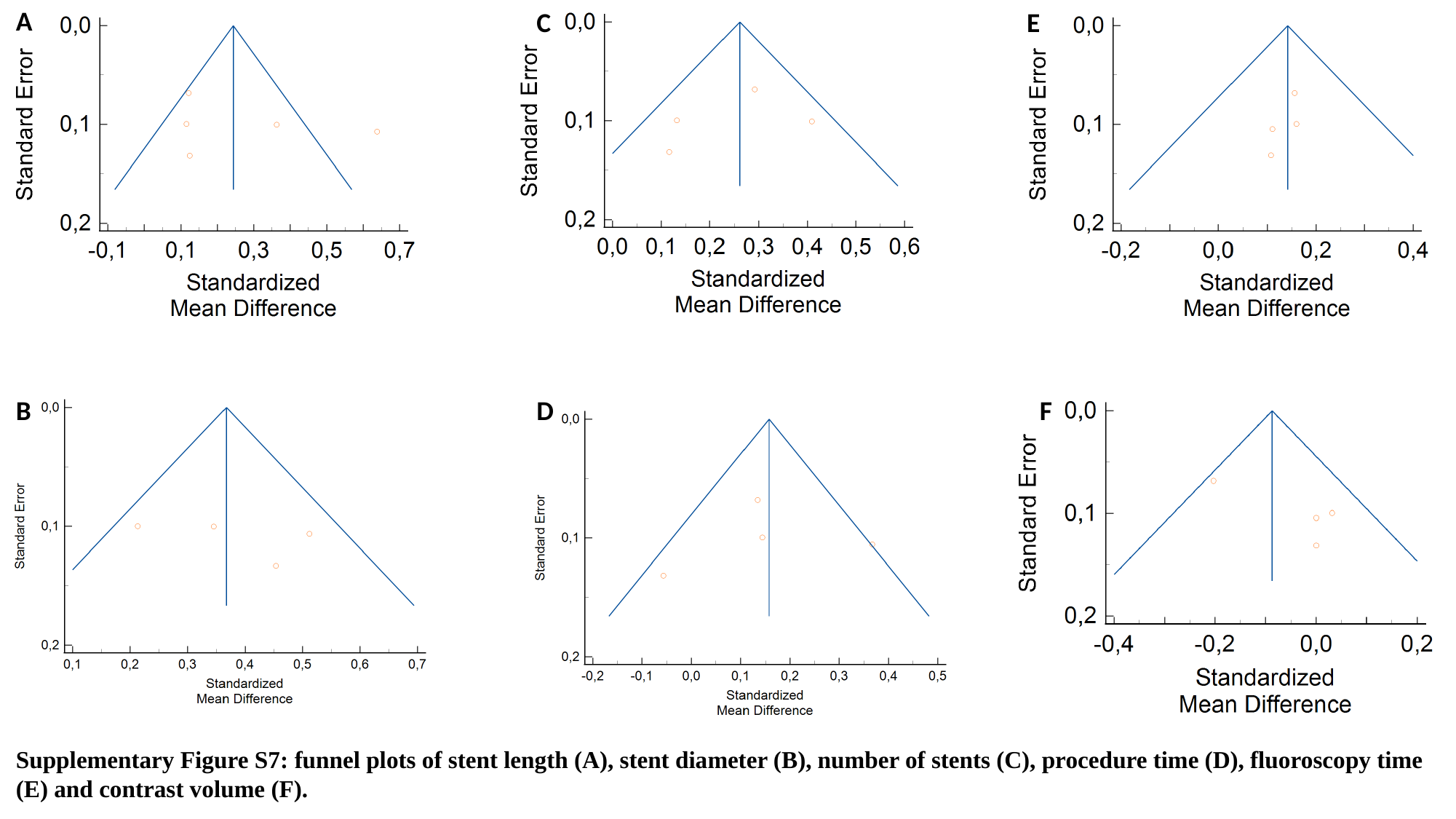

C
E
A
B
D
F
Supplementary Figure S7: funnel plots of stent length (A), stent diameter (B), number of stents (C), procedure time (D), fluoroscopy time (E) and contrast volume (F).
